# Is there room for resilience? A scoping review and critique of substance use literature and its utilization of the concept of resilience

**DOI:** 10.1186/s13011-017-0125-2

**Published:** 2017-09-15

**Authors:** Katherine Rudzinski, Peggy McDonough, Rosemary Gartner, Carol Strike

**Affiliations:** 1 Dalla Lana School of Public Health, University of Toronto, 155 College St, Toronto, ON M5T 3M7 Canada; 2 Centre for Criminology & Sociolegal Studies, University of Toronto, 14 Queen’s Park Crescent West, Toronto, ON M5S 3K9 Canada

**Keywords:** Resilience, Substance use, Drug use, People who use drugs (PWUD), Strengths-based, Risk, Concept analysis

## Abstract

Research in the area of illicit substance use remains preoccupied with describing and analyzing the risks of people who use drugs (PWUD), however more recently there has been a drive to use a strengths-based or resilience approach as an alternative to investigating drug use. This leads us to ask: *what can be known about PWUD from the point of view of resilience?* The objective of this scoping review is to analyze how the concept of resilience is defined, operationalized, and applied in substance use research*.* Popular health, social science, psychology, and inter-disciplinary databases namely: SCOPUS, PUBMED, PsycINFO, and Sociological Abstracts were searched. Studies were selected if they used the concept of resilience and if substance use was a key variable under investigation. A total of 77 studies were identified which provided a definition of resilience, or attempted to operationalize (e.g., via scales) the concept of resilience in some manner. Data were charted and sorted using key terms and fundamental aspects of resilience. The majority of studies focus on youth and their resistance to, or engagement in, substance use. There is also a small but growing area of research that examines recovery from substance addiction as a form of resilience. Very few studies were found that thoroughly investigated resilience among PWUD. Consistently throughout the literature drug use is presented as a ‘risk factor’ jeopardizing one’s ability to be resilient, or drug use is seen as a ‘maladaptive coping strategy’, purporting one’s lack of resilience. Currently, substance use research provides a substantial amount of information about the internal strengths that can assist in resisting future drug use; however there is less information about the external resources that play a role, especially for adults. Though popular, outcome-based conceptualizations of resilience are often static, concealing the potential for developing resilience over time or as conditions change. Studies of resilience among PWUD predominantly concentrate on health-related behaviours, recovery-related factors or predefined harm reduction strategies. Indeed, overall, current conceptualizations of resilience are too narrow to recognize all the potential manifestations of resilience practices in the daily lives of individuals who actively use drugs.

## Background

Research in the area of illicit substance use remains preoccupied with describing and analyzing the deficits, vulnerabilities, and pathologies of people who use drugs (PWUD). This approach is not surprising, since PWUD, like other marginalized groups (i.e., homeless persons, sex workers), are simultaneously constructed as ‘at risk’ in, and ‘a risk’ to, society [1]. Crack use is a case in point. It is commonly perceived as a pervasive criminal and public health problem which has been associated with: physical and mental health consequences (e.g., elevated risks of acquiring HIV and hepatitis C virus (HCV), antisocial personality disorder, major depression); discrimination, stigma, and isolation; and extremely high levels of poverty, homelessness, unemployment and crime [2–11]. Many people who smoke crack likely have experienced turbulent childhoods, high levels of parental drug use, and repeated instances of neglect as well as emotional, physical and/or sexual abuse [4, 12, 13]. Moreover victimization, including physical/sexual assault, robbery, and theft, among individuals who use crack is higher than in the general public, and also often elevated among other drug using groups [14–17].

Although the majority of studies on PWUD examine risk (e.g., [6, 18–21]), more recently there has been a drive to use a strengths-based or resilience approach as an alternative to investigating drug use. Resilience is most commonly defined as ‘positive adaptation despite significant adversity’ [22–24]. Over the years, resilience has been conceptualized in diverse ways: as a trait, as an outcome, and as a process; however, the most common uses of this concept remain outcome-focused [25–29]. Use of resilience terminology in social science research has boomed since the early 2000s [30]. Concurrently, the importance of social and cultural contextualization with respect to resilience also started to be emphasized [24, 31, 32]. Highlighting context is especially important for considering marginalized populations, and uncovering potentially hidden forms of resilience [33, 34].

It is clear that individuals who use crack are exposed to pervasive sources of trauma and adversity, yet there is indication that in spite of such experiences many individuals who use crack find ways to keep going, day after day. The daily hustle to make money, find or maintain housing, and score drugs provides evidence that individuals who use crack are not passive victims of their surroundings, but active participants in difficult circumstances [4, 11, 35, 36]. Research details the ways in which crack-using individuals construct ‘positive’ identities (e.g., mother, hustler, dealer): garnering self-esteem, dignity, and respect for themselves on the streets, as well as taking pride in possessing a repertoire of interpersonal skills and street knowledge which aids their survival in a dangerous social environment [4, 37–40]. Additionally, individuals who use crack are cognizant of the risks of their environment and many make a concerted effort to avoid or minimize risks for themselves in a number of ways (e.g., using alone or with trusted others, leaving dangerous situations) [8, 41–44]. Even among the most victimized crack-using groups, joy in daily successes and hope for future endeavours have been reported [36, 45, 46].

This example highlights the principle that drug use may not be synonymous with a lack of skills or potential. Unfortunately, this is the view most often portrayed in popular culture and academic research alike. Keeping in mind the significant strengths and accomplishments of individuals who use crack, especially under the direct stress and adversity often faced by this group, it is crucial to consider, *what can be known about individuals who use crack, and other PWUD, from the point of view of resilience?* This question provides the impetus for this review.

This scoping review provides an overview and critique of the utilization of the concept of resilience in the substance use literature. Specifically, this review analyzes how the concept of resilience is defined, operationalized, and applied in substance use research, and focuses on the core concepts of resilience research: adversity and risks, internal and external protective factors, and positive outcomes. Applications of resilience as a process, in the recovery field, and among active drug using groups are reviewed separately to highlight these important themes. The goal is to explore the strengths and limitations of the use of this concept, while also stressing significant knowledge gaps. Finally, the value, possible difficulties, and future potential of using resilience perspectives in the study of PWUD are discussed.

## Methods

For this scoping review popular health, social science, psychology, and inter-disciplinary databases, namely: SCOPUS, PUBMED, PsycINFO, and Sociological Abstracts, were searched using various combinations of the terms: resilience, resilient, resiliency, resilience theory, drug use, drug user, substance use, addiction, and dependence. A hand-search of the reference lists of included papers was also conducted. To ensure the most current review of the literature, the focus is placed on studies published within the past 16 years. This is an appropriate temporal scope for review as it coincides with the push for contextualization in resilience research as well as a rise in popularity of the use of this concept in substance use and related fields [24, 30–32]. Studies were chosen if:

1. the term resilience is used, in the title, abstract, or keywords; and,
2. substance use/addiction is a main variable considered in the study.

The focus of this review is primarily on sociological and psychological studies, thus any biological, genetics, and neurosciences research is left out of the analysis. Special care is taken to identify any studies that look at active, illicit drug using populations (e.g., marijuana, cocaine, crack, heroin, opiates, etc.). Studies focusing solely on alcohol or cigarette use are also omitted in order to narrow the scope of this review, and to bring attention to the contentious relationships between illegal drug use and resilience. This review was also limited to studies of PWUD, thus omitting studies on children of drug users. Where inclusion was solely based on evidence of a substance using population, PWUD had to represent a majority, defined as 60% or more, of the population under investigation. Additionally, this review focuses on individual level resilience, where the majority of research is concentrated, rather than family or community-level resilience (e.g., [47–50]). Data were charted and sorted using key terms and fundamental aspects of resilience present in the studies of interest, and reported within the general resilience literature [51]. Based on this, the following themes were derived and will be explored here: definitions and operationalizations, adversity and risk, protective factors, resilience outcomes, process-based conceptualization of resilience, resilience and recovery, and resilience and PWUD.

## Results

A total of 396 studies were retrieved. However, from this group, 319 are excluded because they do not substantially add to the analysis of resilience in the substance use field. Many provide no definition, theoretical backing, or operationalization of resilience (e.g., [52–58]). Others use resilience as a synonym for ‘restraint’ from drug use [59–62], or as a descriptor for individual(s) who adapt well or display toughness [11, 46, 63–65]. The remaining 77 studies selected provide a definition of resilience, or attempt to operationalize (e.g., via scales) the concept in some manner (Table 1).

**Table 1:** Populations of Interest, Scales Used, and Definitions Cited

Citation number	STUDY: Authors, year; (n); Country	Youth	Individuals in treatment/recovery	Active drug users (Majority >60%)	Other information about populations of study	Resilience Scales Used (if any)	Definitions Cited (if any)
[102]	Alavijeh et al., 2016; (*n* = 70); Iran		•		Adult men in a recovery program trial	Connor-Davidson Resilience Scale (CD-RISC)	
[104]	Amandru et al., 2014; (*n* = 200); Uganda	•			Students (14–23 years)	Wagnild & Young Resilience Scale (RS)	Resilience is “a strength that can assist people in positive life adaptation” (Masten and Reed, 2005).
[123]	Andreas et al., 2016; (*n* = 19,303); Norway	•			Middle- & high-school students (mean age 15.4)		
[146]	Barbieri et al., 2016; (*n* = 98); Italy		•		Adult therapeutic community clients	Campbell-Sills & Stein Scale - for resilience at work	Resilience has to do with adversity and positive adaptation (Fletcher and Sarkar, 2013). Resilience comes into play not only in overcoming adversity, conflict, or failure, but also in instances of positive events such as work commitments that require the assumption of new responsibilities.
[157]	Becerra & Castillo 2011; (*n* = 980); Mexico	•			Students (15–22 years)		The ecological risk and resiliency theory examines the relationship between risk and protective factors on individuals within their social contexts (Bogenschneider, 1996; Fraser & Galinsky, 1997; Marsiglia & Waller, 2002).
[126]	Benda et al., 2003; (*n* = 600); US		•		Homeless Vietnam veterans	Specialized scale developed by researchers (5 items)^b^	
[173]	Benda et al., 2005; (*n* = 625); US		•		Homeless Vietnam veterans	Wagnild & Young Resilience Scale	
[79]	Bowland 2015; (*n* = 25); US				Women with histories of trauma		Resilience is defined as “the process of effectively negotiating, adapting to, or managing significant sources of stress or trauma” (Windle 2011, p. 163). Researchers should analyze multiple levels of functioning, including the individual, their life, and their environment, using a human ecology framework.
[148]	Bradshaw et al., 2013; (*n* = 149); US		•		Residential treatment facility participants	Sinclair & Wallston – Brief Resilience Coping Scale	Resilience is an “inner state or intrinsic quality of the human psyche” (Burke, 2006) relevant to the addiction recovery process (Harris et al., 2011). It is the ability to experience pain and difficulty and “snap back” toward an “active process of self-righting and growth” (Higgins, 1994). Resiliency is more than mere belief; it involves self-efficacy and coping skills in the presence of “high-risk” stress and is considered “the core of recovery” (Harris et al., 2011, p. 270).
[140]	Brents et al., 2015; (*n* = 95); US				Adults with childhood experiences of trauma		
[87]	Brothers 2016; (*n* = 30); US			•	Secondary syringe exchangers		Resilience is defined as “the process of harnessing key resources to sustain well-being” (Panter-Brick, 2014, p. 432).
[141]	Brown & Waite 2005; (*n* = 21; *n* = 15); US	•			Resilience education program focus groups with youth & adults		Resiliency factors are defined as strategies used by youth that deter their high-risk behaviors.
[84]	Burnett Jr. et al., 2016; (*n* = 278); US	•			University students (mean age 22)	Wagnild & Young Resilience Scale	Resilience is “a short-term or long-term coping process that has been learned through gradual exposure to progressive challenges and stressors that helps an individual to ‘bounce-back’ with adaptive success” (Richardson, Neiger, Jensen, and Kumpfer, 1990). Resilience is “the ability to maintain relatively stable, healthy levels of psychological and physical functioning”, after exposure to a loss, violence or a life-threatening event (Bonanno, 2004).
[122]	Buttram et al., 2014; (*n* = 562); US *See also Buttram* et al.*, 2014* [213]			•	Sex workers	Pearlin Mastery Scale – self-mastery as proxy	Resilience as measured by personal mastery. Personal mastery (Pearlin & Schooler, 1978) measures the extent to which an individual believes life events or circumstances are under one’s own control.
[172]	Carrico et al., 2015; (*n* = 21); US		•		Men who have sex with men (MSM)		Resilience encompasses the social and psychological resources that assist MSM in effectively coping with social adversity (Herrick, Stall, Goldhammer, Egan, and Mayer, 2014).
[81]	Chang et al., 2003; (*n* = 820); US	•			Incarcerated youth (12–19 years)		Resilience is defined as the ability to be unaffected by, recover from, or acquire strength from adverse life experiences (Carbonell, Reinherz, & Giaconia, 1998). Resilience factors are protective mechanisms that guard those at risk from the effects of adverse life experiences (Rutter, 1987).
[124]	Christiansen & Evans 2005; (*n* = 992); US	•			8th grade students in at-risk urban and rural schools		Resiliency research seeks to discover why some individuals exposed to risk are able to avoid the negative consequences associated with risk exposure (Zimmerman & Arunkumar, 1994). Resiliency theory revolves around understanding the relationships among risk, protective, and outcome variables.
[99]	Cuomo et al., 2008; (*n* = 312 v. *n* = 591); Italy				Incarcerated men	Connor-Davidson Resilience Scale (CD-RISC)	
[118]	Currie et al., 2013; (*n* = 318); Canada			•	Aboriginal adults with illicit & prescription drug problems		Resilience is active in high risk producing conditions, acting to reduce the likelihood of a negative outcome (Johnson et al., 2011; Masten, 2001).
[107]	Daining & DePanfilis 2007; (*n* = 100); US	•			Foster youth (18+ years)		Resilience is defined as a developmental course characteristic of healthy adjustment despite the circumstance of considerable hardship (Luthar, Cicchetti, and Becker, 2000).
[82]	Davis & Spillman 2011; (*n* = 197); US	•			University students (18–44 years)	Specialized scale developed by researchers	Resilience is a “process whereby people bounce back from adversity and go on with their lives. It is a dynamic process highly influenced by protective factors.” (Dyer and McGuinness, 1996, p.276)
[143]	Dell et al., 2005; Canada	•	•		Aboriginals - Hypothetical model/case studies		Resilience is defined as “the extent to which someone can recover from adversity” and describes an individual’s ability to manage or cope with significant adversity or stress in effective ways (Jennison and Johnson, 1997).
[158]	Draper et al., 2015; (*n* = 210); Australia				Older adults (60+) in community health setting	CD-RISC 2 (short)	
[149]	Dufour & Nadeau 2001; (*n* = 20 v. *n* = 20); Canada				Adult women sexually abused during childhood		Resiliency is a person’s ability to return to a previous or even a superior level of adjustment after having experienced a stressful event (Steinhauer, 1998).
[128]	Duque et al., 2013; (*n* = 1780); Colombia	•			Youth (14–26 years) with risk experiences		Resilience is dynamic process molded by culture, where the following factors intervene: making a decision about personal development; what the young person makes of the goods, services, and formal and informal opportunities that are in their reach; and the availability of these (Ungar, 2005).
[86]	Eisen et al., 2014; (*n* = 512); US				Veterans returning home from Iraq & Afghanistan	Bartone Dispositional Resilience (Hardiness) to Stress Scale	Resilience is the ability of adults who are exposed to highly stressful events, such as the violent, life threatening situations encountered in combat, to maintain healthy psychological and physical functioning (Bonanno, 2004).
[96]	Fadardi et al., 2010; (*n* = 120); Iran	•			University students (mean age 21.5)	Connor-Davidson Resilience Scale (CD-RISC)	Resilience is defined as the ability to resist stress and bounce back to normal homeostasis state (Werner, 1986; 2004).
[121]	Gilliard-Matthews et al., 2016; (*n* = 309); US	•			Inner-city African-American and Latino adolescents (13–20 years)		Resiliency theory argues that protective factors in an individual’s social and physical environment aid in their overcoming adverse situations (Egeland, Carlson, and Sroufe 1993; Kaplan et al. 1996). The resiliency process is a complex interconnected system of risks, assets, and resources (Ostaszewski and Zimmerman 2006).
[113]	Gralinski-Bakker et al., 2004; (*n* = 118); US	•			Formerly institutionalized young adults	California Q-Sort on Ego-Resiliency	Resilience has been inferred on the basis of successful adaptation among individuals who faced challenging or threatening circumstances (Luthar et al., 2000; Masten, 2001; Masten et al., 1990; Rutter, 1987). Resilience is empirically defined “in terms of individual outcome profiles encompassing early adult psychosocial development, relationship functioning, and social competence” (Hauser, 1999).
[103]	Green et al., 2014; (*n* = 497); US				Veterans who served in Iraq	Connor-Davidson Resilience Scale (CD-RISC)	Resilience is defined as the capacity to tolerate the effects of trauma exposure or successfully manage following a challenge or setback (Connor and Davidson, 2003). Resilience has been described as a response to situational demands, including the ability to recover from negative and stressful experiences and find positive meaning in seemingly adverse situations setback (Connor and Davidson, 2003; Luthar, Cicchetti and Becker, 2000).
[129]	Griffin et al., 2009; (*n* = 178); US	•			Resilience school programs for middle school youth		Resiliency can be defined as a process of overcoming or averting negative outcomes through the interaction of protective factors and risk factors (Rew and Horner, 2003; Spitler, Kemper, and Parker 2002).
[108]	Hammersley et al., 2015; (*n* = 55); UK		•		Recovering IDUs with experiences of childhood trauma		Resilience explains why some severely traumatised children recover (Cyrulnik, 2009; Werner, 1993). Resilience is created in part by the interaction between the presence of positive social support in the child’s life, and by the child’s ability to elicit support from adults (especially at school).
[142]	Harris et al., 2011; US		•		Hypothetical model/case studies		Resilience is “the community’s inherent capacity, hope, and faith to withstand major trauma, overcome adversity, and to prevail, with increased resources, competence, and connectedness” (Landua, 2007, p. 352). Family resilience is defined as “the path a family follows as it adapts and prospers in the face of stress, both in the present and over time” (Hawley and DeHaan, 1996, p. 293).
[78]	Hills et al., 2016; (*n* = 10); South Africa	•			Street youth (14–18 years)		“In the context of exposure to significant adversity, whether psychological, environmental, or both, resilience is both the capacity of individuals to navigate their way to health-sustaining resources, including opportunities to experience feelings of well-being, and a condition of the individual family, community and culture to provide these health resources and experiences in culturally meaningful ways” (Ungar, 2008, p. 225).
[105]	Hodder et al., 2016; (*n* = 10,092); Australia	•			Students (11–17 years)	The Resilience & Youth Development Module of the California Healthy Kids Survey - protective factors	
[112]	Hollen et al., 2013; (*n* = 243); US	•			Young cancer survivors (14–19 years)		Risk motivation is viewed as a surrogate for resiliency.
[114]	Hopwood & Treolar 2008; (*n* = 8); Australia		•		Hep C treatment clients		Resilience is defined as ‘a class of phenomena characterised by good outcomes in spite of serious threats to adaptation or development’ (Masten, 2001, p. 228). ‘Resilient coping’ is defined as the ability of people to maintain relatively stable and healthy levels of psychological and physical functioning when confronted with a highly disruptive situation (Bonanno, 2004).
[169]	Javdani & Allen 2016; (*n* = 52); US	•			Juvenile justice system involved girls (13–18 years)	Wagnild & Young Resilience Scale (short RS-14)	
[76]	Jones 2012; (*n* = 97); US	•			Foster youth (17+ years) transitioning out of care		Resilience is the ability to make positive adaptations to life’s circumstances despite exposure to severe adversity, and multitude of risk (Luthar, Cicchetti, and Becker, 2000)
[125]	Kassis et al., 2013; (*n* = 5149); Austria, Germany, Slovenia, and Spain	•			Middle school students (mean age 14.5) with history of family violence		Resilience as a holistic concept is better understood if risks are modeled not only on individual factors but also on contextual factors like family and school (Liebenberg and Ungar, 2009; Aisenberg and Herrenkohl, 2008).
[182]	Kidd & Shahar 2008; (*n* = 208); US & Canada	•			Homeless youth (14–24 years)		Resilience can be understood as an ability to mobilize personal and social resources to protect against risks (Rew & Horner, 2003).
[170]	Kurtz et al., 2013; (*n* = 515); US		•	•	An intervention for substance-using men who have sex with men		Resilience focuses on assets and resources to overcome risk (Fergus and Zimmerman, 2005).
[85]	LaFromboise et al., 2006; (*n* = 212); US	•			Aboriginal youth (10–15 years)		Resilience is conceptualized as a protective mechanism that modifies an individual’s response to risk situations and operates at critical points during one’s life (Newcomb, 1992).
[168]	Levey et al., 2016; (*n* = 75); Liberia	•			Youth in post-conflict Liberia (13–18 years)		Resilience is defined as evidence of adaptive functioning and psychological health.
[109]	Longman-Mills et al., 2013; (*n* = 2294); Colombia, El Salvador, Jamaica, Nicaragua, Panama, Uruguay	•			University students with experiences of child abuse		Resilience as the ability to help individuals cope with adversity. Resilience after child maltreatment is aided by biological, social, environmental and psychological factors (Tonmyr, Wekerle, Zangeneh, and Fallon, 2011).
[135]	Luthar & Barkin 2012; (*n* = 827); US	•			Affluent high school youth (11th & 12th grade)		
[174]	Markson et al., 2015; (*n* = 39); UK				Incarcerated men and hardships of reintegration		Resilience is a complex construct (e.g. Cicchetti, 2010; Luthar et al., 2000; Masten, 2007; Rutter, 2012) that covers a ‘reduced vulnerability to environmental risk experiences, the overcoming of a stress or adversity or a relatively good outcome despite risk experiences’ (Rutter, 2012: 336).
[119]	Marsiglia et al., 2002; (*n* = 2125, qualitative *n* = 60); US	•			Latino/a Urban Adolescents (9–18 years)		Resiliency is measured by the degree to which people (or communities) are productive and healthy despite hardships, traumas, and obstacles in their environmental (Bogenschneider, 1996).
[97]	Martin et al., 2014; (*n* = 1149); South Africa	•			High school students with childhood trauma experiences (mean age 16.2)	Connor-Davidson Resilience Scale (CD-RISC)	
[132]	McGloin & Widom 2001; (*n* = 676); US				Adults with childhood abuse/neglect experiences		Resilience as a positive end of adaptation in at-risk samples (Rutter, 1987, 1990). Resilience as good outcomes in spite of high risk, sustained competence under stress; and recovery from trauma (Fraser 1999, Master 1994; Masten 1990).
[150]	McKnight & Loper 2002; (*n* = 355); US	•			Adolescent girls (10–19 years) at risk for delinquency		‘Resilience’ is generally defined as successful coping with or overcoming risk and adversity, the development of competence in the face of severe stress and hardship, and success in developmental tasks or meeting societal expectations, as reflected in overt, behavioural indices such as school grades and ratings by teachers, peers and parents (Doll and Lyon, 1998; Luthar et al., 1993).
[130]	Moon et al., 2000; (*n* = 609); US	•			7th grade students		Resiliency perspective focuses on enhancing those factors thought to protect against or reduce substance use (Norman, 1995).
[181]	Morse et al., 2015; (*n* = 59); UK		•		Art program for addiction recovery service users		
[110]	O’Donnell et al., 2002; (*n* = 2600); US	•			Students (6th, 8th, 10th grade) exposed to community violence		Resilience defined as the ability to cope effectively with stress and to exhibit an unusual degree of psychological strength for one’s age and set of circumstances Werner, 1984). The definition has been expanded in newer studies to include successful coping in specific domains, including both behavioural and emotional arenas (Luthar 1991, 1993; Luthar and Zigler 1991).
[151]	Okamoto et al., 2009; (*n* = 47); US	•			Rural native Hawaiian students (mean age 12.2)		Understand resilience as an outcome of negotiations between individuals and his or her environment (Ungar, 2004). Resilience is socially constructed, contextually specific, and defined by individuals and their social reference group (Ungar, 2004).
[152]	Ostaszewski & Zimmerman 2006; (*n* = 850); US	•			Urban 9th grade students		Resiliency theory emphasizes the role of promotive factors among children growing up in adverse environments, and provides a framework for understanding why some children and adolescents who are exposed to high risk do not develop negative health and social outcomes (Garmezy, 1985; Luthar; 1991; Rutter, 1987).
[180]	Pardini et al., 2000; (*n* = 236); US		•		Adults in recovery from drug and alcohol addiction	Bartone Dispositional Resilience (Hardiness) to Stress Scale	
[136]	Patwary et al., 2012; (*n* = 25); Bangladesh	•		a	Male street youth (20–25 years)		Street competencies can be seen as a positive adaptation to considerable hardships among street people (Luthar, Cicchetti, and Becker, 2000) and terms such as flexibility and resilience may be used to describe homeless youths who are surviving on the street (D’Abreu et al. 1999; Williams et al. 2001).
[171]	Pearce et al., 2015; (*n* = 191); Canada			•	Aboriginal people who use drugs (mean age 28.9)	Connor-Davidson Resilience Scale (CD-RISC)	The most widely accepted definition of resilience in health sciences is positive adaptation despite adversity (Luthar, Cicchetti, and Becker, 2000). A small but growing body of research in Canada has moved beyond individualistic, linear, and western notions of resilience to identify ways in which culture, language, and spirituality buffer adversity and create “cultural resilience” among Indigenous peoples (Fleming and Ledogar, 2008).
[153]	Perkins & Jones 2004; (*n* = 16,313); US	•			Adolescents students (12–17 years)		Resilient people are well-adapted individuals in spite of serious stressors in their lives (Luthar, 1991; Masten, 2001). Human adaptation or competence is composed of the interplay between the context/ecology and the developing organism (Lerner, 1995; Schneirla, 1957).
[117]	Rosenblum et al., 2005; (*n* = 77); US	•			Adolescents (11–15 years) with HIV+ parent		Resiliency is a broad domain that has been variously defined (Greene, 2002; Maluccio, 2002), e.g., capabilities, assets, and positive attributes (Saleebey, 2002); a general frame of reference that guides human beings in coping with environmental challenges (Richman and Bowen, 1997); capacity to rebound from adversity strengthened and more resourceful (Walsh, 1998); and efforts to achieve good developmental outcomes and sustained competence despite the presence of stress and risk (Masten et al., 1990; Werner, 1995).
[77]	Shpiegel 2015; (*n* = 351); US	•			Foster youth (17+ years)		Most scholars currently define resilience as a “pattern of positive adaptation in the context of significant risk or adversity” (Masten and Powell 2003, p. 4). The presence of positive adaptation is generally indicated by (1) achievement of “stage-salient developmental tasks”, or expectations for individual behavior at a specific age; and (2) avoidance of significant psychopathology (Luthar 2006).
[183]	Sirikantraporn et al., 2012; (*n* = 68); US			•	IDUs (18+)		Planning abilities are one of the resilience characteristics of cognitive competence that help individuals achieve their planned goals (Kumpfer, 2002). Resilience development includes positive cognitive abilities that already exist or can be cultivated and strengthened to further increase safe injection practice, decrease risky behaviors, and reduce their chance of contracting HIV/HCV.
[115]	Stajduhar et al., 2009; (*n* = 41); Canada			•	IDUs (and *n* = 45 service providers)		Resilience has been variously defined, but in general, represents a phenomenon involving both adequate and enhanced adaptation in the context of adversity (Roisman, 2005). Resilience includes successful adaptation following a period of maladaptation or developmental difficulty. Indeed, it encompasses not only recovery but includes harm reduction practices.
[179]	Sutherland et al., 2009; (*n* = 128); US		•		Chemically dependent (CD) women in recovery vs. non CD women	Connor-Davidson Resilience Scale (CD-RISC)	Consistently the term resilience has been associated with the ability to recover from adversity. “Resilience describes a process whereby people bounce back from adversity and go on with their lives. It is a dynamic process highly influenced by protective factors” (Dyer and McGuinness, 1996, p. 276).
[120]	Tiet et al., 2010; (*n* = 877); US *See also Tiet & Huzinga 2002* [166]	•			Inner-city youth (longitudinal data set)		Resilience has been defined as having good outcomes despite the exposure to risk (Carlton et al. 2006; Masten 2001; Tiet and Huizinga 2002).
[100]	Tlapek et al., 2016; (*n* = 237); US	•			Child welfare involved female youth (12–19 years)	Wagnild & Young Resilience Scale (short - RS-14)	Resilience was defined as intrapersonal characteristics such as perseverance and self-reliance that allow an individual to adapt to adversity (Wagnild & Young, 1993).
[83]	Tomita 2013; (*n* = 94); Romania				Incarcerated drug using women		The process of, the ability to, or achieving successful adaptation in spite of challenging or threatening circumstances (Masten, Best and Garmezy, 1990).
[116]	Tozer et al., 2015; (*n* = 47); Canada	•		•	Street-involved youth (16–24 years)		Resiliency is perhaps best understood as a person’s ability to navigate and negotiate psychological, social, cultural, and physical resources that sustain their well-being in the context of exposure to significant adversity (Ungar, 2004). Resiliency is as a person’s ability to navigate and negotiate for resources to promote health; however, resources must be accessible and available in order for youth to obtain them (Ungar, 2004)
[154]	Turner et al., 2007; (*n* = 711); US *See also Hartman* et al.*, 2009* [214]	•			High-risk youth (16–23 years)		In spite of the increased likelihood of engaging in delinquency, a significant proportion of individuals, considered to be “high-risk”, prove to be resilient; that is, they overcome the odds and develop into competent human beings (Farrington, Coid, Harnett et al., 2006; Laub and Sampson, 2001; Rutter and Giller, 1983; Smith, Lizotte, Thornberry, and Krohn, 1995; Werner, 1989a).
[111]	Tyler et al., 2014; (*n* = 172); US	•			Homeless youth (19–26 years)		Resilience is generally viewed as having the capacity to overcome serious and cumulative developmental risks to avoid negative outcomes (Rak & Patterson, 1996).
[147]	Veselska et al., 2009; (*n* = 3694); Slovakia	•			8th and 9th grade students (11–17 years)	Specialized scale developed by researchers^c^	Resilience is defined as the process of, capacity for, or outcome of successful adaptation in the face of challenging or threatening circumstances.
[101]	Wachter et al., 2015; (*n* = 191); US	•			Homeless youth (mean age 20.7)	Wagnild & Young Resilience Scale	Resilience is defined as the ability to have a good outcome despite threats to individual development (Masten 2001).
[155]	Waller et al., 2003; (*n* = 32); US	•			American Indian youth (12–15 years)		Resilience is positive adaptation in response to adversity (Waller, 2002). Adversity is typically indexed by two categories of risk factors: (1) challenging life circumstances (e.g., racism, parental drug use, etc.) and (2) trauma (e.g., experiencing family or community violence, death of a parent, etc.; (Masten and Coatsworth, 1998).
[98]	Wingo et al., 2014; (*n* = 2024); US				Adults with childhood experiences of trauma	CD-RISC 10 (short)	Resilience refers to the ability to cope adaptively with adversity or trauma (Luthar, Cicchetti, and Becker 2000). It has been conceptualized as a complex and multidimensional construct with personal characteristics and environmental factors (Feder et al., 2009; Luthar Cicchetti, and Becker, 2000).
[215]	Wong 2008; (*n* = 171); US	•			Middle and high school students (mean age 14.0)		Resilience is positive adaptation in spite of adverse circumstances (Luthar, Cicchetti, and Becker, 2000; Masten, 2001).
[80]	Yates & Grey 2012; (*n* = 164); US	•			Emancipated foster youth (17–21 years)	California Adult Q-Set	Resilience reflects a developmental process wherein the individual is able to utilize resources in and outside the self to negotiate current challenges adaptively and, by extension, to develop a foundation on which to rely when future challenges occur (Egeland, Carlson, and Sroufe, 1993; Yates, Egeland, and Sroufe, 2003) In contexts of prior or current adversity, resilience reflects multiform competence characterized by both the absence of psychopathology and the presence of adaptive capacities to negotiate age-salient issues effectively (Garmezy & Masten, 1986; Luthar, Cicchetti, & Becker, 2000; Masten, 2001).

^a^Authors state that “almost all” participants used drugs without providing numbers

^b^Based on Aroian KJ, Norris AE: **Resilience, stress, and depression among Russian immigrants to Israel**. *Western J Nurs Res* 2000, 22: 54–67

^c^Based on the Resilience for Adults Scale - Hjemdal O, Friborg O, Martinussen M, Rosenvinge J: **Preliminary results from the development and validation of a Norwegian scale for measuring adult resilience**. *J Norwegian Psychol Assoc* 2001, 38: 310–317

The majority of studies focus on youth and their resistance to, or engagement in, substance use. While most of this research focuses on inner-city high school adolescents, studies have also been conducted with affluent youth, young adults in university cohorts, foster youth, and institutionalized adolescents. There is also a small but growing area of research that examines recovery from substance addiction as a form of resilience, and a few studies of unique populations, such as: adults with former childhood experiences of trauma, Aboriginals, veterans, incarcerated individuals, men who have sex with men, street-based individuals, sex workers and PWUD. Several reviews of resilience to drug use among youth were also identified. They are used for further theme development, and cross-referenced to ensure inclusion of all relevant work [66–74].

### Definitions and operationalizations of resilience

Due to the difficulties in defining and operationalizing resilience in the broad literature, it is not surprising that researchers within the field of substance use also experience some complications [24, 27, 75]. This scoping review identified several key types of resilience definitions cited (Table 1). The most commonly cited is the standard definition proposed by Luthar et al. [24] and Masten [22, 23]: ‘resilience is positive adaptation in spite of adversity’. Sometimes the level of adversity is qualified as ‘significant’, ‘severe’, or ‘extreme’ to underline the exceptionally difficult circumstances study participants had to deal with (e.g., physical/sexual abuse, family violence) (e.g., [76–78]).

Another type of definition utilized in the substance use literature is process-based. For example resilience defined as: “the process of effectively negotiating, adapting to, or managing significant sources of trauma” ([79], p. 2). Or in another instance, resilience as “a developmental process wherein the individual is able to utilize resources in and outside the self to negotiate current challenges adaptively and, by extension, to develop a foundation on which to rely when future challenges occur” ([80], p. 472). However, the definition presented and the usages of the concept throughout the study do not always align. For instance, some authors provide a process-based definition, but rely on a trait or outcome-based operationalization with no dynamic elements. These studies put more emphasis on which factors foster resilience, rather than investigate the process of how or why they do (e.g., [81–85]).

Sometimes resilience is defined in relation to the specific outcomes being studied. For instance, Eisen and colleagues [86] define resilience for army veterans as “the ability of adults who are exposed to highly stressful events, such as the violent, life threatening situations encountered in combat, to maintain healthy psychological and physical functioning [including absence of drug use]” ([86], p. 755). Although such limited definitions are clear, their restricted range can sometimes obscure evidence of hidden forms of resilience. Conversely, a more productive approach for defining resilience, especially for marginalized populations, has been to use open-ended definitions (e.g., [79, 87]) which take into account the context and environmental constraints, allowing for more nuanced and hidden forms of adaptation to adversity to be considered as practices of resilience. These types of definitions and their benefits will be taken up in more detail in the *process-based conceptualizations of resilience* and *PWUD and resilience* sections of this review.

The most common operationalization of resilience is as the presence of positive adaptations or absence of negative outcomes. Generally researchers are looking for the absence of drug use which signifies the presence of resilience. While this form of operationalization is direct, it can also be quite restrictive. The various configurations, strengths and drawbacks of outcome operationalizations will be discussed in the *resilience outcomes* section.

Scales are also often utilized to operationalize resilience in the substance use field, most commonly versions of the Connor-Davidson Resilience Scale (CD-RISC) or the Wagnild & Young Resilience Scale (RS) (Table 1). Although scales are a straightforward and consistent approach to operationalizing resilience, many are overly focused on individual level traits, characteristics, and skills. For example, common items include ego strength, sense of purpose, goal-orientation, self-efficacy, self-esteem, hardiness, tenacity, self-mastery, optimism, spirituality/faith, adaptive coping, problem-solving skills, cognitive flexibility, educational expectations, empathy, and sense of humour [88–94]. Although the CD-RISC includes aspects such as secure relationships and knowing how to facilitate social support, while the RS considers an individual’s need for social approval, companionship and assistance, both of these scales are still primarily focused on individual-level factors [88, 92, 93, 95]. So essentially when utilizing such scales researchers are putting considerable weight on the individual-level aspects of resilience, and performing trait-based examinations. As such, they leave out much, or any, notion of other salient factors (e.g. external resources, environmental circumstances) that could affect measurements of resilience.

Interestingly the majority of authors who adopt a trait-focused conceptualization of resilience rely on resilience scales (e.g., [84, 96–103]). They account for a third of all scale usage in this scoping review. In contrast, a number of authors recognize the limitations of trait-focused resilience scales in capturing the concept holistically, and adapt by measuring external protective factors as well. For instance, in addition to using the RS, Amandru et al. [104] also look at the effects of social support, which include informational, tangible and affectionate support as well as positive social interactions with family and peers. Another exception to these trait-focused scales is Hodder and colleagues’ [105] study of resilience to drug use among students, where the researchers make use of the Resilience and Youth Development module of the California Healthy Kids Survey, which measures both internal assets and external resources, such as family, school and community engagement.

Resilience research is wrought with definitional and operationalization difficulties. This concept is typically operationalized through outcomes measuring the occurrence of positive, or the lack of negative, adaptations. When process-based definitions are provided their usage of the concept throughout the research does not always align. Scales are also often utilized to operationalize resilience, yet many are overly focused on individual level factors. Many authors who subscribe to a trait-focused conceptualization of resilience rely on resilience scales, while others recognizing the limitations of this approach adapt by measuring external factors as well.

### Adversity and risk as precursors of resilience

A necessary antecedent of resilience is adversity, with researchers often asking the important question: *resilience with respect to what?* [106]. This scoping review found three main categories of adversity: traumatic events, disease processes, and daily stressors. Traumatic events include physical, sexual, and emotional abuse, childhood maltreatment/neglect, violence, and criminal victimization (e.g., [78, 79, 84, 97, 98, 100, 107–111]). Disease processes under investigation encompass addiction, mental health problems, HCV/HIV progression, and cancer diagnosis (e.g., [112–117]). Daily stressors often overlap with other risk factors considered, and comprise conditions or experiences such as living in high risk neighbourhoods, poverty, homelessness, discrimination, school problems, family discord, and transitioning out of foster care (e.g., [76, 77, 85, 87, 111, 118–122]). Drug use is often considered a daily stressor or risk factor for resilience for the individuals under investigation (e.g., [80, 123–126]).

The intensity and duration of adversity exposure is crucial when studying resilience [30, 127], yet only a few studies explicitly measure the levels of adversity and differences in exposure between study participants. For example, O’Donnell et al. [110] differentiate between students who witness community violence and those who are personally victimized, when measuring their respective risk for drug involvement. Meanwhile, Duque and his fellow researchers [128] ascertain a minimum level of risk exposure among Colombian youth, by defining a resilient youth as: “one that has experienced *three or more* risk factors but has not presented any of the severe aggressive behaviors or any of the other risky behaviors… [including drug use]” ([128], p. 2212).

In contrast to these studies, several do not explain how adversity is measured. This type of ‘taking adversity for granted’ occurs most often in studies of student drug use, where the reader is left to assume that the threat facing students is the social influence to try drugs [82, 96, 105, 129]. The dilemma with this approach is that since it is not measured how often, if ever, these youth are offered drugs, it is “impossible to assess whether or not these students had to bounce back from adversity and go on with their lives” ([82], p. 18). This is not the case in all student studies. For instance, Moon et al. [130] attempt to quantify the ‘threat of drug offers’ for students by looking at the event from several key vantage points. These researchers ask youth about ever having been offered drugs, the context of the last offer (i.e., location of offer: school, party, park, street, friend’s home, own home), and the age of first use. Likewise, Andreas et al. [123], in their study of ‘who says no to cannabis offers’, consider three groups of students: cannabis users, cannabis naïve (those who have never received an offer to use) and cannabis resilient (those who decline offers for cannabis); thereby expanding the research of protective factors beyond the overly simplistic dichotomy of cannabis user vs. non-user. Nonetheless, the threshold for adversity in resilience research continues to be a point of contention [131, 132]. As Fletcher et al. [131] explain, some researchers state that adversity must include a significant negative life event(s), known to be “statistically associated with adjustment difficulties” ([133], p. 858), while others view adversity less strictly, allowing it to encompass any type of hardship, misfortune or difficulty [131, 134].

Although it is very important for authors to be clear about the adversity facing their populations of interest, some researchers take this to the extreme and continue to over-focus on risk and adversity (e.g., [83, 108, 135, 136]). For example, Tomita’s [83] study of drug using women prisoners claims to look at protective factors to improve resilience and reintegration into the community after release. However, a considerable portion of the paper categorizes and discusses the various risk factors for addiction these women face inside and outside of prison. Resilience on the other hand, is presented as a future goal, defined and contextualized in previous research, but not used to analyze the current data. Thus, resilience in some of these risk-focused studies is treated as an adjunct or after-thought rather than being utilized to its full potential as a concept.

This scoping review found that drug use is often treated as a daily stressor or risk factor for resilience. Only a few studies investigate the intensity and duration of adversity exposure. A disagreement concerning the necessary level of adversity continues in the literature whereby, some investigators only focus on resilience to major life misfortunes, while other researchers investigate evidence of resilience to any type of difficulty. Problematically, a number of researchers take adversity for granted, typically in student studies where the assumption is: adversity is the negative social influence to try drugs.

### Internal and external protective factors

Protective factors are internal strengths and external resources that interact with risks to affect the chances of negative outcomes for individuals [69, 137, 138]. In this field, a substantial number of researchers continue to see resilience as an “inner state or intrinsic quality of the human psyche” ([139], p. 284), with some authors emphasizing the psychological and personality aspects of resilience against drug use (e.g., [96, 111, 112, 140]), while others discuss the need for a resilient constitution or skill set to resist involvement with drugs (e.g., [73, 102, 109, 141]). In essence, when focusing on individual-level factors, resilience is presented as an attribute that ‘must be’ developed (e.g., through resilience education/school programs) or maintained (e.g., throughout addiction recovery) [66, 129, 142].

Commonly considered internal factors include: self-esteem, self-efficacy, personal skills (e.g., coping, problem solving, social, help seeking), intellectual ability, religiosity/spirituality, and optimism, (Table 2). One unique, drug-specific internal factor considered is ‘attitudes about drug use’, which focuses on such elements as: fear of consequences of drug use (i.e. health problems, parental disapproval), belief that drugs will interfere with one’s future goals, anti-drug personal norms, and no interest in drug use [82, 112, 117, 119, 121, 123, 141].

**Table 2:** Internal Protective Factors Studied

STUDY	Self-esteem	Self-worth/ self-respect	Self-efficacy	Personal skills	Intellect/Knowledge	Self-control/personal mastery	Religiosity/spirituality & cultural identity	Optimism/hopefulness	Autonomy/agency	Personality traits/attitudes
Alavijeh et al., 2016 [102]				•		•				
Andreas et al., 2016 [123]										•
Barbieri et al., 2016 [146]				•				•		•
Benda et al., 2003 [126]	•		•		•		•			
Benda et al., 2005 [173]	•		•							
Bowland 2015 [79]				•			•			
Bradshaw et al., 2013 [148]								•		
Brents et al., 2015 [140]										•
Brothers 2016 [87]			•	•						
Buttram et al., 2014a [122]					•	•				
Carrico et al., 2015 [172]				•						
Chang et al., 2003 [81]	•				•			•		
Currie et al., 2013 [118]	•			•			•		•	
Daining & DePanfilis 2007 [107]							•			
Davis & Spillman 2011 [82]										•
Dufour & Nadeau 2001 [149]	•								•	
Eisen et al., 2014 [86]				•						
Fadardi et al., 2010 [96]			•			•				
Gilliard-Matthews et al., 2016 [121]										•
Gralinski-Bakker et al., 2004 [113]	•	•	•							•
Griffin et al., 2009 [129]				•						
Hammersley et al., 2015 [108]			•					•		
Hills et al., 2016 [78]				•		•	•	•		
Hollen et al., 2013 [112]					•	•				•
Hopwood & Treolar 2008 [114]				•			•	•		
Javdani & Allen 2016 [169]			•	•						•
Jones 2012 [76]				•	•			•		
Kassis et al., 2013 [125]		•		•		•		•		
Kidd & Shahar 2008 [182]	•									
Kurtz et al., 2013 [170]				•						
LaFromboise et al., 2006 [85]	•						•			
Levey et al., 2016 [168]	•			•		•	•	•	•	
Longman-Mills et al., 2013 [109]							•			
Marsiglia et al., 2002 [119]										•
Martin et al., 2014 [97]				•						
McKnight & Loper 2002 [150]			•				•			
Morse et al., 2015 [181]	•	•	•	•				•		
Okamoto et al., 2009 [151]							•			
Ostaszewski & Zimmerman 2006 [152]		•	•	•			•	•		
Pardini et al., 2000 [180]							•	•		
Patwary et al., 2012 [136]				•						
Perkins & Jones 2004 [153]							•	•		
Rosenblum et al., 2005 [117]		•	•	•	•	•				•
Shpiegel 2015 [77]					•		•	•		
Sirikantraporn et al., 2012 [183]				•	•					
Stajduhar et al., 2009 [115]		•	•	•		•		•	•	•
Sutherland et al., 2009 [179]						•				
Tozer et al., 2015 [116]		•	•							•
Turner et al., 2007 [154]	•	•			•		•			
Tyler et al., 2014 [111]			•				•			•
Veselska et al., 2009 [147]	•									
Wong 2008 [215]						•				
Yates & Grey 2012 [80]	•			•	•					

There is value in highlighting the inner strengths of individuals in overcoming adversity as, for example, Dell et al. [143] do so by discussing strengthening the inner spirit and cultural connectedness of Aboriginal youth overcoming solvent addiction. The problem is that not all researchers discuss how resilience may be strengthened or weakened by interactions (and choices) between the individual and their potential external resources [108]. Moreover, a restrictive emphasis on individual-based resilience can lead to some problematic dichotomies. For instance, ‘resilient’ individuals are seen as having ‘what it takes’ to refrain from drug use, while those ‘non-resilient’ persons who ‘succumb’ to addiction are seen as weak, deficient and blameworthy [37, 144, 145]. These types of studies reinforce dominant negative opinions of PWUD and, as Kassis et al. [125] warn, perpetuate further ‘victim-blaming’.

Numerous studies on substance use utilize the term ‘resiliency’, often interchangeably with ‘resilience’ (e.g., [76, 82, 100–102, 109, 111, 112, 119, 121, 124, 129, 130, 141–143, 146–155]. This inconsistent use of terminology occurs in spite of multiple warnings from long-time resilience researchers, that choosing ‘resiliency’ terminology suggests an intrinsic quality of the person rather than the achievement of positive outcomes based on various levels and interactions of protective factors and resources [23, 24, 132]. Additionally, resilience is often equated with protective factors in the substance use literature. The term ‘resilience factor’ is often used interchangeably with ‘protective factor’ (e.g., [81, 109, 117, 118, 147, 150]), further adding to the conceptualization of resilience as a static factor(s), rather than a dynamic concept dependent on both protective and risk elements. Given the fact that the field of resilience research is already fraught with definitional and operationalization difficulties, consistency or, at minimum, clarity in the use of terminology within and across disciplines is crucial.

Moving beyond internal traits and characteristics, many studies in the field of substance use also consider external resources for resilience, at three broad levels: family, school and community. Frequently explored external protective factors at the family level are: parental supervision, family management (e.g., setting boundaries and appropriate consequences) family support (e.g., trust, adaptability, and cohesion), family bonding (e.g., closeness, communication, and cultural ties), and support from a partner. At the school level researchers look at: positive school environment, good relationships with teachers, school engagement, involvement in extra-curricular activities, and positive peer connections. Finally, at the community level community engagement, supportive relationships with friends or community members (e.g., neighbours, case workers), participation in religious/spiritual practices, and formal community supports (e.g., social services, addiction programs, housing) are considered (Table 3).

**Table 3:** External Protective Factors Studied

STUDY	Family Level					School Level			Community Level					OTHER
	Parental supervision & monitoring	Family management (setting boundaries, appropriate consequences)	Family support (trust, adaptability, cohesion)	Family bonding (closeness, communication, cultural ties)	Supportive relationships with partner	Positive school environment (supportive relationships with teachers)	School engagement (commitment, sense of belonging, extra- curricular activities)	Positive peer connections	Community engagement (commitment, sense of belonging, caregiving)	Supportive relationships with friends	Supportive relationships with community members (neighbour, case worker)	Participation in religious/ spiritual activities	Formal Supports (drop-in programs, treatment, work, housing, etc.,)	
Amandru et al., 2014 [104]			•	•				•		•	•			
Andreas et al., 2016 [123]	•	•		•										
Barbieri et al., 2016 [146]									•				•	Positive work environment; social support at work
Becerra & Castillo 2011 [157]	•		•	•										
Benda et al., 2003 [126]			•	•	•					•		•	•	
Benda et al., 2005 [173]			•	•	•					•				
Bowland 2015 [79]									•		•	•	•	
Bradshaw et al., 2013 [148]			•											
Brothers 2016 [87]											•	•	•	
Buttram et al., 2014a [122]			•	•	•					•	•		•	
Carrico et al., 2016 [172]													•	
Chang et al., 2003 [81]	•		•											
Christiansen & Evans 2005 [124]	•			•		•		•	•	•	•			
Currie et al., 2013 [118]														Enculturation: Aboriginal cultural participation
Daining & DePanfilis 2007 [107]			•		•					•	•			
Davis & Spillman 2011 [82]	•	•	•	•				•		•				
Draper et al., 2015 [158]									•	•			•	
Dufour & Nadeau 2001 [149]			•	•										
Eisen et al., 2014 [86]			•	•	•					•	•			
Gilliard-Matthews et al., 2016 [121]	•							•						Neighbourhood safety
Griffin et al., 2009 [129]											•		•	
Hammersley et al., 2015 [108]			•				•				•			
Hills et al., 2016 [78]										•			•	
Hopwood & Treolar 2008 [114]			•		•					•			•	
Javdani & Allen 2016 [169]							•				•		•	
Jones 2012 [76]			•	•						•	•		•	
Kassis et al., 2013 [125]	•					•		•						
Kidd & Shahar 2008 [182]										•	•			
LaFromboise et al., 2006 [85]			•	•					•					Enculturation; neighbourhood safety
Levey et al., 2016 [168]			•				•							
Luthar & Barkin 2012 [135]	•	•		•										
Markson et al., 2015 [174]			•	•	•								•	
Marsiglia et al., 2002 [119]	•	•	•	•		•								
McKnight & Loper 2002 [150]			•	•		•	•							
Morse et al., 2015 [181]										•				Visiting new places
Moon et al., 2000 [130]				•								•		Neighbourhood safety
O’Donnell et al., 2002 [110]	•			•		•	•	•		•				
Okamoto et al., 2009 [151]			•	•										
Ostaszewski & Zimmerman 2006 [152]			•	•				•		•		•		
Pardini et al., 2000 [180]			•		•					•	•	•		
Patwary et al., 2012 [136]										•				
Pearce et al., 2015 [171]				•									•	Enculturation
Perkins & Jones 2004 [153]			•	•		•	•	•			•	•		
Rosenblum et al., 2005 [117]				•									•	Neighbourhood safety
Shpiegel 2015 [77]			•				•				•			
Sirikantraporn et al., 2012 [183]													•	
Stajduhar et al., 2009 [115]			•				•			•	•		•	
Sutherland et al., 2009 [179]			•		•						•	•	•	
Tiet et al., 2010 [120]	•	•	•	•		•	•							
Tomita 2013 [83]		•		•										
Tozer et al., 2015 [116]			•							•	•		•	Drug use by family & friends; stigma & group norms; responsibilities for others; fear of losing family/friends; enculturation
Turner et al., 2007 [154]			•	•		•						•		
Tyler et al., 2014 [111]												•		
Waller et al., 2003 [155]			•	•										
Wong 2008 [215]	•		•	•										
Yates & Grey 2012 [80]				•	•				•	•	•			

Social support in all its forms (informational, material, and emotional) is a key external factor for resilience, found across all three abovementioned broad levels of external protective factors (e.g., [82, 85, 86, 104, 110, 122, 153]). Indeed, social connectedness (e.g., care, trust, attention, and shared time) and interpersonal relationships feature significantly as external protective factors in many of the reviewed studies (e.g., [76, 79, 104, 113, 119, 124]). However, in this literature a significant amount of attention is paid to family-level and school-based external protective factors. This is most likely due to the abundance of youth studies.

Environmental and structural protective factors are less commonly invoked in studies of resilience and drug use. For instance, Longman-Mills et al. [109], in their study of university students who had a history of childhood maltreatment, attempt to bring such factors to the forefront by considering the child protection laws in each of the Latin American and Caribbean countries where they carried out their research. However, these structural factors are not considered in the final analyses.

Religiosity is an interesting protective factor as it features in both internal and external domains. Sometimes this factor is considered an indication of individual faith/spiritual strength (i.e., *“how important are religious beliefs to you”* ([109], p. 81), versus religion as an external factor which encompasses elements such as devotion rituals and congregation participation (e.g., [79, 130]). Meanwhile, some authors combine internal and external measures such as the importance of spirituality and the level of service attendance, reporting it as one variable (e.g., [111, 126, 153, 154]). Similarly, culture is also a protective factor that spans the realm of internal strengths (i.e., cultural values) and external resources (i.e., cultural practices, enculturation) (e.g., [85, 118, 143, 151]).

Internal protective factors are a significant focus in many substance use papers. Researchers continue to see resilience as a trait, emphasizing personality aspects or skills that help people to resist drugs. The focus is on changing the individual, rather than changing the resources available, for that individual, to strengthen resilience. This can perpetuate victim-blaming. Inconsistent use of terminology creates added confusion for concept definitions and operationalizations. External resources for resilience are examined to a lesser extent by research studies. Social support is a key factor for resilience that is found across multiple levels of external resources.

### Resilience outcomes

Positive adaptations are generally considered consequences of resilience for individuals facing adversity. The most common conceptualization of resilience in the substance use literature is outcome-based. This is not altogether surprising, as use of this concept remains consistently outcome-focused throughout many other fields as well (e.g., [28, 75, 156]). However, whether trait, outcome, or process-based conceptualizations of resilience, or a mix of these approaches, are used, more often than not, substance use is rendered a maladaptive behaviour, and the ways in which some individuals are able to resist or remain ‘resilient’ from substance use is a key line of inquiry in the field of addictions.

Many studies in the reviewed works employ a simple approach, where the presence of resilience is judged by one single outcome measure: the absence of substance use or abuse (e.g., [82, 96, 98, 104, 105, 109, 112, 118, 119, 121, 123, 130, 140, 147, 151, 152, 155, 157, 158]). Resilience here is understood as the capacity to avoid or withstand using drugs, and consequently PWUD are depicted as non-resilient, again giving rise to problematic dichotomies. It is interesting that some researchers do not clarify whether they are concerned with the absence of any level of substance ‘use’, or if the emphasis is more specifically on ‘problem’ drug use or abuse (e.g., [71, 100]). Given that among youth some level of drug experimentation is normal during this period of development, and that desistance in drug use often occurs by a natural process of ‘ageing out’ [159–161], studies that classify drug using youth as lacking resilience are setting a detrimental precedent. Certainly, this is a problem with outcome-based conceptualizations of resilience in general, as they tend to be static in nature and obscure the possibility of individuals developing resilience to certain outcomes over time, or as circumstances change [75, 137].

Meanwhile, other studies use a multi-dimensional approach to investigate the consequences of resilience, focusing on the presence of several pre-determined positive adaptation measures and/or the absence of negative adjustments (Table 4). Often substance use is examined alongside various other anti-social conduct measures that need to be avoided to provide evidence of resilience: alcohol and cigarette use, mental health problems, risky sexual activity, homelessness, aggression/violence, delinquency/criminal involvement, and conduct problems (school) or rule breaking. In terms of pre-determined positive adaptations, researchers commonly consider: educational attainment, employment, marriage, good interpersonal relations and social skills, good health, and psychosocial functioning. Many of these positive adaptations are biased (i.e., white, middle class) in that these “traditional markers of functionality” ([80], p. 488) often exclude or do not adequately capture resilience experiences for marginalized populations [162, 163].

**Table 4:** Social Deviance and Positive Functioning Indicators Studied in Addition to Drug Use

STUDY	Social Deviance Indicators								Positive Functioning Indicators				
	Alcohol and/or cigarette use	Mental health issues	Risky sexual activity	Homeless-ness	Aggression/ violence	Delinquency/ criminal activity	Conduct problems/ rule breaking	Other	Educational attainment/ competence	Employment	Marriage	Good relations /social skills	Other
Andreas et al., 2016 [123]	•	•			•	•		Impulsivity					
Barbieri et al., 2016 [146]										•			
Becerra & Castillo 2011 [157]	•												
Brown & Waite 2005 [141]			•										
Burnett Jr. et al., 2016 [84]	•												
Carrico et al., 2015 [172]			•										
Daining & DePanfilis 2007 [107]				•		•		Early parenthood	•	•			
Draper et al., 2015 [158]	•												
Duque et al., 2013 [128]			•		•								
Eisen et al., 2014 [86]	•	•											
Gralinski-Bakker et al., 2004 [113]		•				•			•	•	•	•	Social and leisure activities; parenting
Green et al., 2014 [103]		•						Suicidal ideation					
Griffin et al., 2009 [129]	•				•								
Hammersley et al., 2015 [108]						•		Abusive relationships					
Hollen et al., 2013 [112]	•												
Javdani & Allen 2016 [169]	•	•	•		•	•		Somatization					Self-efficacy
Jones 2012 [76]				•		•			•	•	•		Future optimism; independent living skills
Kidd & Shahar 2008 [182]								Hopelessness; loneliness; suicidal ideation					Good health
Kurtz et al., 2013 [170]			•					Sensation seeking					
LaFromboise et al., 2006 [85]	•				•	•	•		•				
Levey et al., 2016 [168]			•										Daily functioning; realistic goals for the future
Longman-Mills et al., (2013) [109]	•												
Luthar & Barkin 2012 [135]	•	•					•	Somatization					
Markson et al., 2015 [174]	•			•		•				•		•	Good health; coping ability
McGloin & Widom 2001 [132]		•		•	•	•			•	•		•	
McKnight & Loper 2002 [150]	•					•							
O’Donnell et al., 2002 [110]	•	•				•	•	Somatization				•	Future optimism; self-reliance
Ostaszewski & Zimmerman 2006 [152]	•												
Perkins & Jones 2004 [153]	•		•					Purging; attempted suicide	•				Helping others
Shpiegel 2015 [77]		•		•		•		Teenage pregnancy	•				
Tiet et al., 2010 [120]		•			•	•	•	Gang Involvement; isolation; cruelty^a^	•				Self-esteem
Tlapek et al., 2016 [100]	•	•						Revictimization					
Turner et al., 2007 [154]						•							
Tyler et al., 2014 [111]						•							
Veselska et al., 2009 [147]	•												
Wingo et al., 2014 [98]	•												
Wong 2008 [215]	•						•		•				

^a^Based on The Child Behavior Checklist (CBCL) - Achenbach, TM, Edelbrock, CS. Manual for the Child Behavior Checklist and revised child behavior profile. 1983. Burlington: University of Vermont, Department of Psychiatry

By expanding their view of potential outcomes, researchers are able to acknowledge that the concept of resilience is multi-dimensional in nature, in that an individual who struggles in one domain can simultaneously possess strengths in another/others [24]. Using such an approach is less likely to lead to problematic dichotomies common with single-domain resilience measures. In fact, some researchers are able to move beyond the extremes of resilient vs. non-resilient categories and instead apply multi-level classifications. As a case in point, Daining & DePanfilis [107] who studied foster youth transitioning into adulthood, consider resilience as the result of a composite score (from 0 to 12) across six domains of functioning (i.e., presence of educational and employment participation, as well as avoidance of early parenthood, homelessness, drug use and criminal activity), allowing for low, medium, and high resilience classifications among participants. Alternately, McGloin & Widom [132], in their study of abused and neglected children grown up, examine eight functionality domains (i.e., employment, education, and social activity attainment, in addition to absence of psychiatric disorder, homelessness, substance abuse, criminal arrest and violence), and consider a score of 6 out of 8 to demonstrate resilience. These examples reveal that even among studies which investigate several domains of functioning some, like the latter example, will continue resorting to binary categorizations of resilient vs. non-resilient, while others, like the former, allow for a potentially more inclusive operationalization, recognizing the existence of some level of resilience in each category.

Markedly with multi-domain approaches to resilience, some studies allow for low-levels of anti-social behaviour, including drug use. For instance, low level drug use results in lower scores for that domain, but leaves the potential open for better scores in other domains (e.g., [76, 77, 107, 120]). Some investigators recognize the importance of examining both internal and external realms of functioning, seeing the potential for an individual to be internally resilient (e.g., have high levels of psychosocial functioning and self-esteem) and/or externally resilient (e.g., perform well academically, sustain employment and avoid drug use, gang involvement and delinquent activities) (e.g., [80, 120]). However, there is some debate about whether internal and external resilience should be recognized independently, or whether only those individuals who possess both internal and external adaptation should exclusively be considered resilient [164–166].

In the substance use literature outcome-based conceptualizations of resilience are most commonly utilized. Many studies judge the presence of resilience by the absence of substance use or abuse. Some studies use multi-dimensional approach adjudicating presence of resilience based on several pre-determined positive adaptation measures or on the absence of negative adjustments. Problematically, outcome-based conceptualizations of resilience are often stagnant and can conceal the potential for developing resilience over time, or as conditions change. However looking at resilience from a multi-dimensional perspective holds great promise, especially if the domains are selected in the context of what is socially and culturally relevant for the population under investigation.

### Process-based conceptualizations of resilience

Braverman ([167], p. 4) argues that “[resilience] researchers have thus far been more successful in identifying protective factors than in explaining how they operate”. This definitely rings true in the substance use literature, where significantly fewer researchers attempt to conceptualize resilience as a dynamic process, which explores the interactions between risk and protective factors, than those who utilize an outcome or trait-based approach to operationalize resilience [79, 86, 87, 102, 108, 110, 113, 115, 120, 124, 129, 151, 152, 154, 155, 168–172]. Just under half of these papers are qualitative investigations of resilience, while the rest are quantitative, utilizing resilience models and longitudinal data sets.

In a qualitative study of adult women trauma survivors in low-income housing, Bowland ([79], p. 2) considers resilience as a dynamic process, and cites an open definition of the concept: resilience is “the process of effectively negotiating, adapting to, or managing significant sources of stress or trauma”. By investigating resilience in context, taking into account the individuals, their lives and the constraints of their environment, this researcher is able to effectively analyze practices of resilience for her study population. Indeed, Bowland considers both instances of isolation and experiences of community engagement to be examples of resilience in the midst of adversity. Such a fluid approach to resilience can be quite useful for examining this concept in vulnerable populations.

The fundamental focus in qualitative studies on how risk and protective factors interact with one another, allows for appreciating the dual nature of some of these factors, i.e., seeing how they act protectively in some circumstances and as risks in others. Okamoto and colleagues [151] find that in some cases, both risk and protection occurs because of the close knit family networks of Hawaiian youth. For example, certain family members can protect individuals from drug offers, while it is particularly those close familial bonds that make it hard to refuse drug use in other instances.

When considering substance use and resilience among populations of inner-city/urban adolescents, surveyed longitudinally, both Ostaszewski & Zimmerman [152] and Tiet et al. [120] utilize dynamic models. Using their data, Ostaszewski & Zimmerman [152] test the compensatory and risk-protective models of resilience, finding support for the former model and discovering that processes of resilience may operate more effectively at high levels of risk. This study also acknowledges the important cumulative effects of protective factors. Tiet et al. [120], on the other hand, develop their own conceptual models which assess interactions of risk and protective factors, helping the authors predict changes in resilience over time. This study provides another prime example of how factors can have a dual nature. In the models tested by Tiet and colleagues [120], over time self-esteem is positively correlated with better adjustment, predicting good psychosocial functioning and academic achievement. Yet, self-esteem is also found to predict higher levels of anti-social behaviour (e.g., drug use, gang involvement). Tiet and his fellow researchers muse that perhaps it takes a certain level of self-esteem for youth to have the courage and charisma to engage in deviant activities and be accepted by delinquent peers.

Although a number of researchers support process-based definitions of resilience, many studies, nevertheless, produce static and outcome-focused conceptualizations of resilience (e.g., [85, 128, 132]). However, several researchers stress the necessity to conceptualize resilience as a process in future studies and advocate for future longitudinal research in order to “capture constructs over time”, examining specific interactions between risks, protective factors, resilience, and drug use (e.g., [80, 107, 167]). Yet it must be emphasized that not all longitudinal studies automatically consider resilience as a process (e.g., [173, 174]). Benda [173], in his longitudinal study of homeless veterans who abuse substances measures resilience at only one point in time. Thus, the investigator is unable to provide any information on the changes in resilience over the lives of the study participants.

Fewer studies use a process-based approach to investigate resilience. This dynamic approach to resilience research often uses open-ended definitions which can be quite useful for examining resilience in vulnerable populations. Looking at interactions between risk and protective factors allows us to appreciate the dual nature of these factors, acting protectively in some circumstances and as risks in others. Although a number of researchers support process-based definitions of resilience, many studies, nevertheless, produce static and outcome-focused conceptualizations.

### Recovery as resilience

There is a debate in resilience research about the proper definition of this concept, with many researchers, especially those within the substance use literature, choosing to restrict resilience to individuals who never give in to risks or exhibit maladaptive behaviours (e.g., substance use, addiction) [137, 175]. Meanwhile, there are a smaller number of researchers who hold a wider view of resilience, considering recovery as a distinct example of this concept in action [115, 176, 177]. Thus, when drug-using groups are looked at with respect to resilience, the predominant trend is to look at PWUD in recovery or treatment (Table 1). In fact, many studies focus on aspects such as the process of natural recovery, inner strength, or substance use interventions/treatments, leaving out any notion of the resilience involved in persevering in one’s daily life with an ongoing addiction. A special issue on resilience in the journal *Substance Use & Misuse* is devoted primarily to examining recovery or the development of children with alcoholic or drug using parents [178].

Definitions of resilience in papers on recovery commonly focus on ‘bouncing back’ from adversity, with addiction considered the most prominent form of adversity [126, 143, 148, 179]. Drug use is often portrayed as a maladaptive coping strategy, and resilience is defined as being able to stay on the path toward recovery from addiction [142, 179]. Maintaining long-term sobriety is considered a positive outcome in these studies [102, 148, 180]. Harris et al. [142], in their Process Model of Addiction and Recovery, stress the idea of ‘resilience to relapse’ by stating that the ‘compulsive cycle of substance dependence’ shows a lack of resilience, while the ‘coping cycle of recovery’ demonstrates resilience in action.

A few of studies comparing resilience between in-treatment/recovery/institutionalized PWUD and ‘healthy’ non-users were also found. These studies consider drug abuse as a maladaptive coping strategy and the majority use scales to measure resilience, finding that addicted individuals consistently score lower on these instruments [99, 179]. Dufour and Nadeau [149] underscore the anti-thesis between resilience and addiction, while investigating mental health among women who had been sexually abused during childhood. From the outset these researchers designate one group as ‘resilient’ and the other as ‘addicted’, failing to consider any evidence of resilience among the in-treatment women. This is especially surprising given the authors’ comments that the ‘addicted’ women are interviewed at the beginning of recovery, “at a moment where they have chosen to help themselves in getting treatment, at a time when they felt a bit more in control of their destiny” ([149], p. 667).

Such restrictive dichotomies are prevalent throughout this area of research (e.g., [108, 148]). Dell et al. [143] argue for the need to reconsider defining individuals who return to active substance use as treatment ‘failures’ with respect to resilience. Yet, this continues to be the underlying assumption, partly due to the over emphasis on individual-level protective factors, (e.g., readiness to change, motivation, determination, hardiness, psychological strength, spirituality, and optimism), often featured in recovery research [102, 114, 142, 148, 172, 179, 180]. It seems that in some ways resilience is co-opted as yet another trait that helps people recover, and differentiates those individuals from persons who continue to use drugs.

The key to a more holistic and contextualized approach to resilience research may be to provide equal attention to external protective factors (e.g., social support, family assistance, community programs, enculturation), and consider the mechanisms and interactions between the individual and their potential resources through which resilience can be built/maintained [108, 143, 146]. In fact, resilience-based recovery programs and interventions, focusing on both internal strengths (e.g., confidence, inner spirit, pride, creativity, developing new skills) and external resources (e.g., community supports, social interaction, cultural participation, aftercare), are currently being developed and evaluated [102, 143, 170, 172, 181].

When resilience is researched among PWUD the predominant trend is to examine individuals in recovery or treatment. Under these circumstances drug use is usually considered a maladaptive coping strategy, and resilience is identified as maintaining long-term sobriety. Individuals who relapse back into active substance use are deemed as ‘failures’ with respect to resilience, partly due to the over emphasis of individual-level protective factors. Resilience is often designated as a trait that differentiates recovering individuals from persons who actively use drugs.

### PWUD and resilience

In this scoping review of substance use literature, only a small number of studies were found that fully invoked the concept of resilience to examine behaviours of individuals who currently use drugs (Table 1). Several resilience studies discussed current drug use among homeless youth (e.g., [78, 101, 111, 136, 168, 182]), however most had no clear indication of how many study participants were actively using. Moreover, the focus of these studies remains on dealing with the adversity of the street, and/or on revealing which protective factors are associated with a reduction or cessation of drug use, as well as a number of other social deviance indicators (e.g., criminal involvement, prostitution, suicidal ideation). Drug use is typically portrayed as a habit that needs to be supported, a risk factor for individuals on the street, or a maladaptive coping strategy at odds with resilience.

In this section, the focus of the analyses will be restricted to studies which clearly state that a majority (>60%) of participants are PWUD. Among these studies there is a shift from regarding ‘any’ drug use as a negative outcome, to only regarding ‘problem’ drug use (e.g., dependence, heavy use, drug use before/during sex) in this manner [116, 118, 170]. This type of approach broadens who can be considered resilient, from non-users to PWUD who are not high-risk. For instance, Tozer and colleagues [116] investigate elements associated with the prevention of transitioning to injection drug use among street-involved youth. In this study, youth who avoid transition to injection are deemed resilient, thus positioning injection drug use specifically, rather than drug use generally, as the negative outcome under consideration.

This scoping review found only five studies that specifically recruited PWUD and utilized the concept of resilience [87, 115, 122, 171, 183]. Two of these studies use scales to measure resilience, with both trying to moderate the drawbacks of these standard instruments, which are often not able to capture significant aspects associated with resilience for marginalized populations. Buttram et al. [122] in their study of substance-using sex workers utilizes a proxy measure of resilience, self-mastery, yet also measures external factors (i.e., health insurance, transportation access, and social support) in trying to discern what reduces syndemic risk factors (including drug use) for this population. Meanwhile, Pearce and colleagues [171] utilize the CD-RISC to measure resilience, in a study that examines risk and protective factors associated with resilience among a cohort of young Aboriginal peoples who use illicit drugs. However, to strengthen the subjective dimensions of their resilience research, these researchers add elements of cultural connectedness and traditional language, allowing them to see other protective resources for their population. In this study resilience is postulated as resistance to detrimental health outcomes, specifically HCV and HIV, and drug use is considered a risk factor for these health outcomes, yet it is one among many risk factors.

The remaining three studies in this section examine resilience among people who inject drugs (PWID). Generally these studies concentrate on health-related behaviours and harm reduction strategies as evidence of resilience. For example, Stajduhar et al. ([115], p. 310) attempt to reconceptualise resilience for a population of PWID by providing a ‘wider’ definition: “resilience includes successful adaptation following a period of maladaptation or developmental difficulty. Indeed, it encompasses not only recovery but includes harm reduction practices”. However, evidence of resilience, is researcher-defined and remains recovery-related (i.e., quitting or decreasing use), with secondary emphasis on harm reduction strategies (i.e., support seeking or safer use). Moreover, continued drug use as a coping strategy, for these researchers, is considered suppressed resilience. Likewise, Sirikantraporn et al. [183], focus entirely on researcher pre-defined harm reduction behaviours, such as ‘planning to ensure steady access to clean injection equipment’, as evidence of resilience among PWID. Although both of these studies point to the unexplored potential of resilience research among PWID and to the power of using this approach to uncover strengths and ‘safer’ drug use practices that promote well-being, the present conceptualizations remain too narrow to see all the potential manifestations of resilience.

Despite these continuously restricted conceptualizations of resilience, researchers who examine this concept among PWUD are able to show that many protective factors identified in the resilience literature for other populations can also be found for individuals who currently use drugs (Tables 2 and 3). Moreover, a few researchers are able to demonstrate that a lot of what would be deemed risk factors can actually have desired outcomes, in terms of resilience, for these vulnerable populations [115, 116]. For instance, Tozer et al. [116] highlight several untraditional protective factors for young non-injectors, including how the presence of adult injectors or injection by family members, two factors often considered risky, can provide protection in cases when adult injectors discourage injection or when youth see the consequences of injection for family members. By considering the social context of protective factors these researchers are able to emphasize varying sources of support and constraints in the lives of their study participants.

In a novel approach to operationalizing resilience among PWID, Brothers ([87], p. 2) in her study of secondary syringe exchangers (SSEs) relies on an open definition of resilience as “the process of harnessing key resources to sustain well-being”. Building on from this Brothers recognizes ‘the syringe’ as the resource being utilized by SSEs, through their practices of collecting used syringes, exchanging them at harm reduction agencies for new syringes, and distributing clean syringes to other PWID. These acts of exchange in the context of a risk environment are considered by Brothers as examples of resilience in practice, as SSEs work to improve their financial and mental well-being. This approach to resilience among PWUD supports the important notion that these individuals are active agents who have significant strengths and capabilities that should be recognized as manifestations of resilience.

Very few studies were found that thoroughly investigated resilience among PWUD. These studies predominantly concentrate on health-related behaviours, recovery-related factors or pre-defined harm reduction strategies. Drug use is still often considered a negative outcome or risk factor; however some studies attempt to differentiate between ‘any’ drug use and ‘problem’ drug use. Although these studies endeavour to broaden the concept of resilience for PWUD, providing important information regarding protective factors for these individuals, nevertheless overall these conceptualizations are still too limited to recognize all the potentially hidden forms of resilience in the daily lives of individuals who actively use drugs.

## Conclusions

The most common operationalizations of resilience in substance use literature are outcome-based, focusing on the presence of positive adaptations (e.g., abstinence, recovery) or absence of negative outcomes (e.g., drug use, relapse). However this scoping review reveals that there are ongoing difficulties in the utilization of the concept of resilience in this field. Confusion in operationalization and lack of clarity around whether resilience is being treated as a trait, outcome or process (e.g., definitions do not align with conceptualization; reliance on scales over-focused on individual-level factors) as well as issues with inconsistent use of terminology (e.g., resilience vs. resiliency; protective vs. resilience factors) have resulted in a field of study where it is difficult to compare, contrast, and build upon current research. Researchers who do not articulate what they mean by adversity, protective and risk factors, and positive outcomes further add to the semantic ambiguity.

Currently, substance use research provides a substantial amount of information about the internal strengths that can assist in resisting future drug use; however there is less information about the external resources that play a role, especially for adults. Though popular, outcome-based conceptualizations of resilience are often static, concealing the potential for developing resilience over time. Indeed using a dynamic process-based approach, which relies on open-ended definitions or looks at changes in resilience over time, can provide a more useful method for examining resilience in vulnerable populations. At present, when resilience is studied among PWUD the predominant trend is to examine individuals in recovery/treatment. In the few instances when resilience is investigated among persons who are currently actively using drugs the focus remains on health-related behaviours, cessation-related factors (quitting or decreasing use) or predefined harm reduction strategies. Although such progress is encouraging, the present uses of the concept of resilience still disregards resilience practices involved in persevering in one’s daily life with an ongoing addiction.

The concept of resilience, as it is currently utilized, is too restrictive and, in essence, leaves drug using individuals out of the realm of investigation [162, 184], leading to a dearth of studies on resilience among PWUD. Consistently throughout the literature, substance use is presented as the antithesis of resilience: drug use is seen as a ‘maladaptive coping strategy’, purporting one’s lack of resilience, or drug use is presented as a ‘risk factor’ jeopardizing one’s ability to be resilient. Problematically many studies fixate on individual-level assets as markers of resilience, most commonly relying on scales and failing to consider wider environmental resources [92, 95]. However, resilience scales, which are often created for white, middle class populations, may not capture features significant for more marginalized groups, resulting in consistently lower scores for PWUD [99, 179]. Similar issues have historically been raised with IQ scales and quality of life measures (e.g., [185–190]). This restricted view of resilience is not surprising given the presiding prohibitionist culture where drug use is prohibited, PWUD are punished, and abstinence is considered the best policy [191]. The strength of these underlying ideas is apparent, when considering the public and political controversy caused by harm reduction initiatives in Canada in recent years (e.g., distribution of safer crack use kits, operation of safe consumption sites) [192–196]. Thus, it is no wonder that it is so hard for academics, and the general public alike, to accept that PWUD have the potential for resilience, at least while their use is ongoing.

It is clear from the current use of resilience in studies of PWUD that researchers have only begun to carve out the necessary room for inclusion. Most studies focus on recovery as a form of resilience, which although a step in the right direction, has definite drawbacks as well. The road to recovery is incredibly difficult and often fraught with adversities, thus the value of shedding light on the resilience of individuals who work towards this goal is indispensable. However, what must be reiterated is that recovery should not be considered the sole form of resilience available for PWUD. The problem lies in the fact that recovery from addiction is adjudicated on separate and highly moral grounds. For instance, the recovery model for mental health problems considers success as attaining an “improved quality of life within the limitations caused by the presence of illness” ([181], p. 233), whereas success for addiction recovery is measured by a significant reduction, if not a total abstinence from drug use [197]. Since recovery is typically synonymous with abstinence from drug use, acknowledging this as the only practice of resilience open to PWUD essentially corroborates the limited view that resilience is the absence of drug use, espousing a viewpoint that already dominates in this literature. However, the literature on recovery from addiction accepts the view of “recovery as a process”, which involves growth, set back, and fluctuations [198–200], thus it may be open to regarding resilience as modifiable, allowing for process-based conceptualizations for this phenomenon.

Part of the issue with the lack of resilience studies among PWUD is that this concept is difficult to define and operationalize for this ostracized population. Often what is needed to ‘do well’ or ‘get by ‘in a drug environment (e.g., tough persona, street knowledge, drug-involved social network contacts) is not considered valuable in other contexts (e.g., school, workplace). Fergus and Zimmerman (2005) argue that resilience processes can include a ‘lack of dysfunctional’ outcomes; however, it is telling that most studies in the substance use literature focus on this absence of ‘negative’ outcomes which does not automatically indicate the presence of ‘positive’, fulfilling lives among participants [69, 201]. Consistently associating drug use with other socially unacceptable consequences leads to this behaviour being regularly interpreted as a risk factor, and omits the reality that many individuals use drugs for various self-perceived benefits in the context of their daily lives [70]. A contextualized approach to analyzing resilience that addresses internal assets, skills and capacities, especially as perceived by the individuals themselves, as well as considers currently accessible resources and external dynamics in the family, community and structural environment (e.g., group supports, social policies), is crucial for recognizing potentially ‘hidden’ forms of resilience among marginalized populations [32, 163, 202].

In spite of the current difficulties, this scoping review recognizes several important strengths of utilizing the concept of resilience for studying substance use. Firstly, a multi-dimensional conceptualization of resilience creates a more inclusive approach, and in some studies allows for low levels of drug use, without immediately categorizing PWUD as non-resilient. This is a crucial step to seeing resilience among PWUD. However, what is missing is an in-depth evaluation of which other dimensions, beyond typical normative functionality assessments (e.g., academic success, employment), marginalized populations can identify with. A more open-ended definition of resilience, found in several qualitative studies (e.g., [79, 87]), allows for precisely such an investigation. Additionally, these studies show that, such an unstructured approach to resilience research makes it possible to uncover unique responses and adaptations, which can signify resilience in a given context. Finally, process-based conceptualizations of resilience are beneficial in that they concentrate on the manifestations of resilience, that is on the ways in which individuals display resilience (i.e., how individuals use internal strengths or external resources), rather than focusing on measuring ‘levels of resilience’ (e.g., scale measures, domain scores), as outcome studies do. This kind of exploration provides a better basis for understanding what is really crucial during times of adversity, in order to increase the potential for resilient outcomes for a particular group.

Within the last decade, parallel research fields (e.g., homelessness, sex work) have begun to recognize the potential resilience has to assist in understanding the practices involved in negotiating adverse circumstances among such marginalized populations (e.g., [203–207]). Likewise, there has been a drive to extend who can be considered resilient in the substance use realm. Researchers looking at PWUD strive to show how potential manifestations of resilience are present. Currently these types of analyses have mainly limited themselves to focusing on health-related outcomes, emphasizing reductions in use, motivations for recovery, and safer use practices. However, by engaging the possibility for resilience among individuals who continue to use drugs, these studies provide another significant step to creating the space necessary for furthering resilience research among PWUD.

In fact, the concept of resilience holds incredible potential for research among PWUD. Currently, studies of persons who actively use drugs showcase the possibilities of resilience research and the power of using this line of inquiry to examine unexplored strengths and ‘safer’ drug use practices that promote well-being for PWUD [115, 183]. Although conventional conceptualizations of resilience are strained when applied to the study of marginalized populations, this can motivate researchers to engage a “more contextually sensitive understanding of resilience”, which takes into account environmental constraints, available resources, and the values and goals of individuals under investigation [32, 79, 87, 101]. Such an approach provides us with a “corrective lens” when analyzing seemingly ‘anti-social’ practices of PWUD, or other vulnerable populations, allowing for a deeper awareness of how these behaviours are appropriate expressions of resilience in the given context [101, 208, 209]. Indeed, the concept of resilience, despite its many complexities, gives investigators a platform from which to highlight hidden, socially marginalized, misunderstood, or de-valued practices indicative of resilience, and to explore the unique ways in which individuals appraise, adapt to, and cope with different forms of adversity in a variety of social and cultural contexts [184, 210].

Resilience research among active drug using populations has the potential to dispel some of the myths and negative stereotypes regarding PWUD. Returning to our introductory example of crack smoking; currently there persists a particularly negative discourse around crack use and people who use crack. In fact, perhaps more so than any other addict, the crack user has been ‘vilified and ostracized’ in both popular culture and academic research [37, 211, 212]. By shifting focus to the strengths and capacities of individuals who use crack, this kind of research can promote a view of these individuals as capable, resourceful, motivated persons who persevere amidst difficult circumstances. Research among individuals who use crack can lend credibility to the resilience practices that these individuals engage in by documenting them as well as using the information gained to advocate on behalf of this population. Such research is also crucial for creating new policies, programs, and interventions that can benefit PWUD.

## Abbreviations

CD-RISC: Connor-Davidson Resilience Scale
HCV: hepatits C virus
HIV: human immunodeficiency virus
IQ: intelligence quotient
PWID: people who inject drugs
PWUD: people who use drugs
RS: Wagnild & Young Resilience Scale
SSEs: secondary syringe exchangers
